# The Transcription Factor Cux1 Regulates Dendritic Morphology of Cortical Pyramidal Neurons

**DOI:** 10.1371/journal.pone.0010596

**Published:** 2010-05-11

**Authors:** Ning Li, Chun-Tao Zhao, Ying Wang, Xiao-Bing Yuan

**Affiliations:** 1 Institute of Neuroscience and State Key Laboratory of Neurobiology, Shanghai Institutes for Biological Sciences, Chinese Academy of Sciences, Shanghai, China; 2 Graduate School of the Chinese Academy of Sciences, Chinese Academy of Sciences, Shanghai, China; 3 School of Lab Medicine and Life Science, Wenzhou Medical College, Wenzhou, China; Medical College of Georgia, United States of America

## Abstract

In the murine cerebral cortex, mammalian homologues of the Cux family transcription factors, Cux1 and Cux2, have been identified as restricted molecular markers for the upper layer (II-IV) pyramidal neurons. However, their functions in cortical development are largely unknown. Here we report that increasing the intracellular level of Cux1, but not Cux2, reduced the dendritic complexity of cultured cortical pyramidal neurons. Consistently, reducing the expression of Cux1 promoted the dendritic arborization in these pyramidal neurons. This effect required the existence of the DNA-binding domains, hence the transcriptional passive repression activity of Cux1. Analysis of downstream signals suggested that Cux1 regulates dendrite development primarily through suppressing the expression of the cyclin-dependent kinase inhibitor p27^Kip1^, and RhoA may mediate the regulation of dendritic complexity by Cux1 and p27. Thus, Cux1 functions as a negative regulator of dendritic complexity for cortical pyramidal neurons.

## Introduction

The Cux (also known as Cut and CDP) proteins are a family of homeobox transcription factors identified in all metazoans and implicated in the regulation of cell proliferation and differentiation in many organisms (reviewed in reference [1], [2]). In higher vertebrates, two Cux genes, Cux1/CDP and Cux2, have been identified [3], [4], [5]. The murine Cux1 is expressed in most tissues, including the brain [6], while Cux2 is enriched primarily in the nervous system [5]. In the mouse cerebral cortex, both Cux1 and Cux2 are expressed in postmitotic pyramidal neurons of upper cortical layers from embryonic stages to adulthood and in precursor cells of the proliferative ventricular and subventricular zones (VZ/SVZ) [7], [8], [9], [10]. However, the function of Cux genes in the mammalian central nervous system remains largely unknown. Cux2 was reported to control the proliferation of neural progenitor cells at the cortical SVZ area and the spinal cord, although with opposite effects in these two regions [11], [12]. Beyond the regulation of cell proliferation, the persistent expression of Cux genes in postmitotic pyramidal neurons in upper layers of the cortex suggests that they may have specific functions in these differentiated cells [8], [10].

Cortical pyramidal neurons have well-defined dendritic trees [13]. Considering that Cut regulates the dendritic branching patterns of *Drosophila* multidendritic da sensory neurons [14], we examined the potential function of Cux proteins in the development of dendritic trees of cortical pyramidal neurons. We found that Cux1, but not Cux2, could regulate the dendritic complexity of cultured cortical pyramidal neurons. The expression of the cyclin-dependent kinase inhibitor, p27^Kip1^, was specifically suppressed by Cux1, and co-expression of p27 with Cux1 could compensate Cux1's effect on dendritic arborization of cortical pyramidal neurons. Furthermore, the small GTPase RhoA may mediate the regulation of dendritic complexity by Cux1 and p27. Thus, our results indicate that Cux1 can regulate dendritic morphology of cortical pyramidal neurons.

## Results

### Cux1 can regulate dendritic morphology of cultured cortical pyramidal neurons

**Figure 1:**
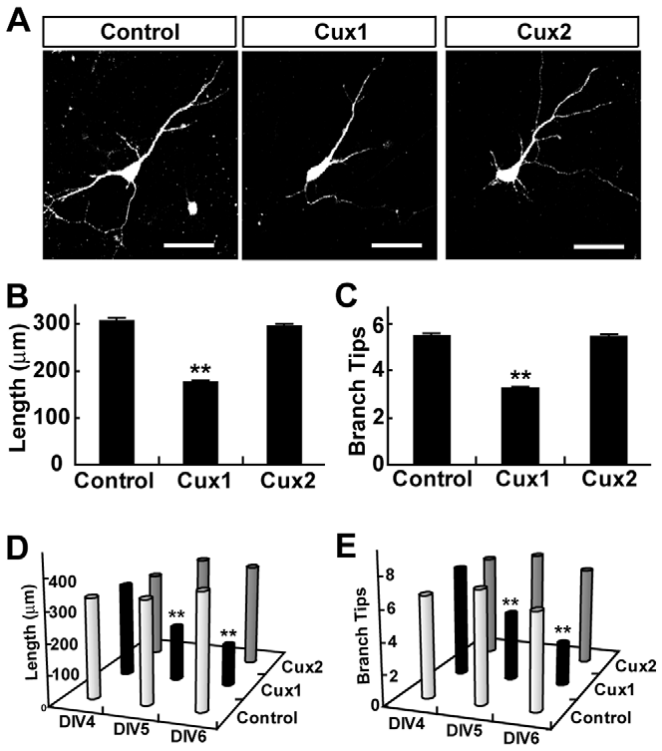
Expression of Cux1, not Cux2, simplifies the dendritic morphology of cortical pyramidal neurons in culture. (A) Representative examples of transfected neurons at DIV6. Scale bars: 50 µm. (B–C) The dendritic morphology of transfected cells at DIV6 was measured by Length (B) and Branch Tips (C). Data shown are mean ± SEM (n≥120 for each condition). ** *P*<0.01, Student's *t* test. (D–E) Time-course of dendritic complexity change after transfection. Time points examined were DIV4, 5, and 6 (n≥30 for each condition). Data are shown in 3-D column view. ** *P*<0.01, Student's *t* test.

To examine the function of Cux proteins in postmitotic cortical pyramidal neurons, we cloned the Cux1 and Cux2 genes from the rat brain and transiently transfected them into cortical neurons after culturing *in vitro* for 3 days (DIV3). Comparing to cells transfected with the control vector, Cux1-overexpressing pyramidal neurons showed a reduced dendritic complexity (Figure 1A). At DIV5 and DIV6, the dendritic arborization of Cux1-tansfected cells was markedly reduced, as indicated by the total dendritic branch length (Length; Figure 1B, D) and the total dendritic branch tip number (Branch Tips; Figure 1C, E). The dendritic complexity was not significantly changed at DIV4 (Figure 1D–E), suggesting that the regulation of dendritic complexity by Cux1 is not a fast process. In contrast to Cux1, the dendritic complexity of Cux2-transfected cells was not affected at all time points examined (Figure 1). Together, these data showed that increasing the intracellular level of Cux1, but not Cux2, could reduce the dendritic complexity of cortical pyramidal neurons in culture.

**Figure 2:**
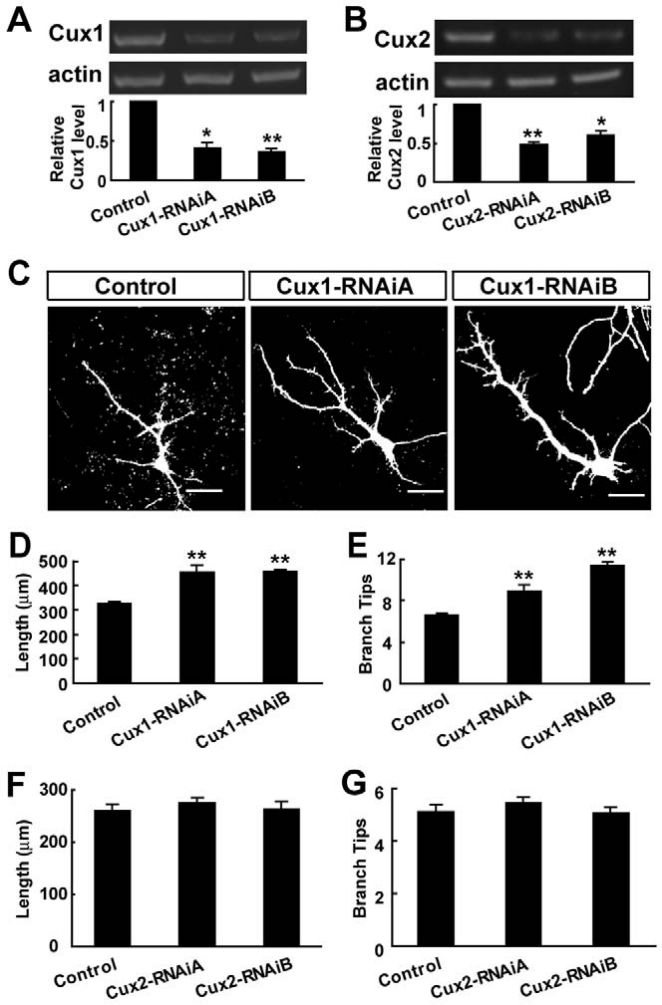
Down-regulation of Cux1 by RNAi increases the dendritic complexity of cortical pyramidal neurons. (A–B) RT-PCR analysis of Cux expression level following RNAi treatments. Two specific RNAi each designed to different regions of ORF for Cux1 and Cux2, Cux1/2-RNAiA and -RNAiB, could result in the down-regulation of their expression levels. Representative images are semi-quantitative PCR. Quantitative results are measured by real-time PCR. Data shown are mean ± SEM (n = 3 independent experiments). * *P*<0.05, ** *P*<0.01, paired *t* test. (C–E) Increase of pyramidal neuron dendritic complexity after transfection of Cux1-RNAi plasmids. Representative cells are shown in (C). Scale bars: 50 µm. Data shown are mean ± SEM (n≥30 for each condition). ** *P*<0.01, Student's *t* test. (F–G) Dendritic morphology of pyramidal neurons after transfection of Cux2-RNAi plasmids. Data shown are mean ± SEM (n≥30 for each condition). *P*>0.05, Student's *t* test.

To further examine the function of Cux1, we designed two RNA-interference (RNAi) constructs targeting different regions of the Cux1 ORF. Consistent with previous reports [15], [16], both of the RNAi constructs, Cux1-RNAiA and Cux1-RNAiB, could effectively down-regulate Cux1 expression (Figure 2A). When we transfected the RNAi constructs into cortical pyramidal neurons in culture, they increased the complexity of dendritic arborization (Figure 2C–E). In contrast, RNAi-knockdown of Cux2 did not affect dendritic arborization of cortical pyramidal neurons in culture (Figure 2B, F, G). Therefore, Cux1 has a regulatory effect on the dendritic morphology of cortical pyramidal neurons.

### Transcriptional repressor activity of Cux1 is required in regulating dendritic morphology

**Figure 3:**
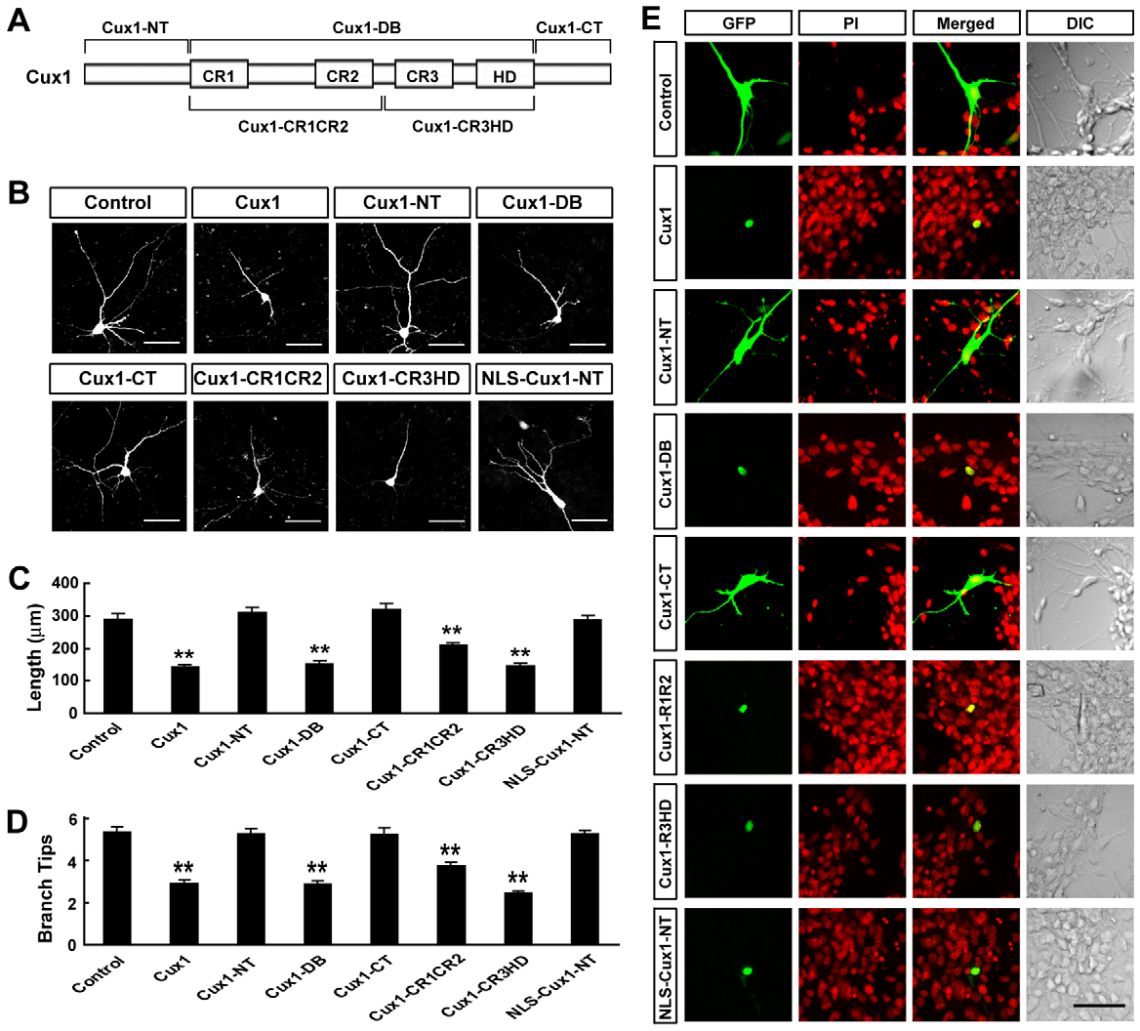
Domain analysis of Cux1's effect on dendritic complexity of cortical pyramidal neurons. (A) Domain structure of Cux1 protein. Constructed deletions of Cux1 are indicated. (B) Representative cells after transfection of different deletions of Cux1. Scale bars: 50 µm. (C–D) Quantification of the dendritic complexity for cells transfected with various Cux1 deletions. Data shown are mean ± SEM (n≥30 for each condition). ** *P*<0.01, ANOVA SNK test. (E) Localization of Cux1 deletions in cortical pyramidal neurons in culture. Cux1 and Cux1-deletions were tagged with GFP to reveal their subcellular localization. Propidium iodide (PI) was used to indicate the nuclei. DIC: differential interference contrast. Scale bar: 50 µm.

Cux1 is generally regarded as a transcriptional repressor with four conserved DNA-binding domains: three Cut repeats (CR1 to CR3) and one Cut homeodomain (HD) (Figure 3A). To examine whether transcriptional repressor activity of Cux1 is required to regulate dendritic morphology of cortical pyramidal neurons, we constructed three different deletions of Cux1: Cux1-DB contained all four DNA-binding domains, while Cux1-NT and Cux1-CT referred to the remaining N- and C-terminal fragments, respectively (Figure 3A). Comparing to the control, Cux1-NT and Cux1-CT did not reduce the dendritic complexity of cortical pyramidal neurons in culture (Figure 3B–D). In contrast, overexpressing Cux1-DB resulted in a significant decrease in dendritic complexity, comparable to the effect of the full-length Cux1 (Figure 3B–D). In addition, we constructed Cux1-CR1CR2 and Cux1-CR3HD, each has half of the four DNA-binding domains (Figure 3A). Both deletions could decrease the complexity of dendritic arborization (Figure 3B–D). We noted that all Cux1 deletions could be appropriately expressed and localized to the cell nucleus or cytoplasm (Figure 3E). To eliminate the possibility that the subcellular localization rather than the DNA-binding activity is required, we added nuclear localization signal (NLS) to Cux1-NT, which effectively drove the expression of Cux1-NT into the nucleus (Figure 3E). However, this construct could not affect the dendritic arborization (Figure 3B–D). These results suggest that DNA-binding domains, hence the passive repression activity, is required for Cux1 to control the dendritic morphology of cortical pyramidal neurons.

### p27 is required for Cux1 to regulate dendritic morphology

**Figure 4:**
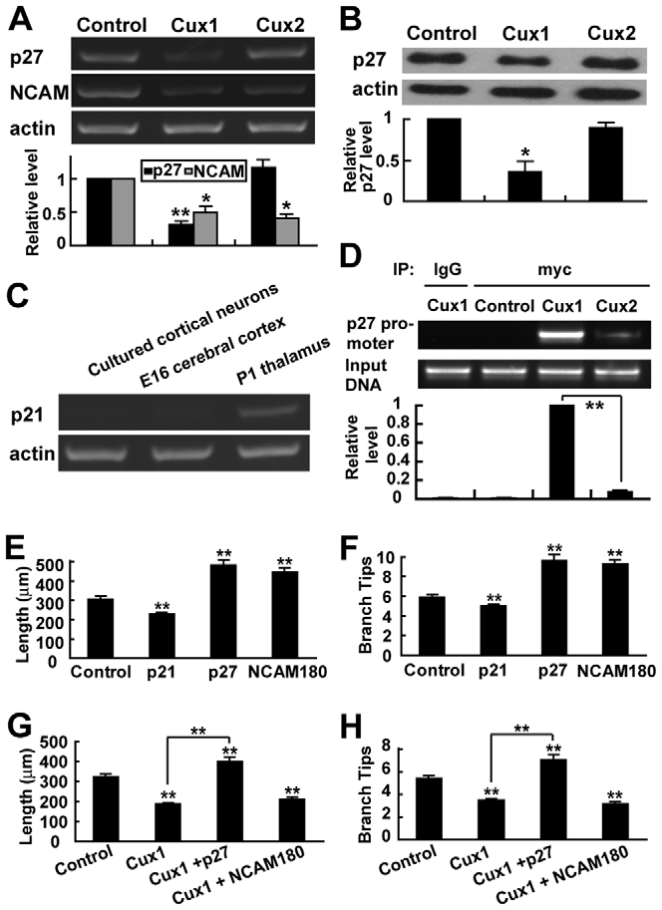
p27 is required for Cux1 to regulate dendritic morphology of cortical pyramidal neurons. (A) RT-PCR analysis of p27 and NCAM expression levels after overexpression of Cux proteins. Representative images are from semi-quantitative PCR. Quantitative results are measured by real-time PCR. Data shown are mean ± SEM (n = 3 independent experiments). * *P*<0.05, ** *P*<0.01, paired *t* test. (B) Western blotting of p27 protein level following overexpression of Cux proteins. Data shown are mean ± SEM (n = 3 independent experiments). * *P*<0.05, paired *t* test. (C) RT-PCR analysis of p21 expression level in cultured cortical neurons and E16 cortical tissue. P1 thalamus tissue was used as a positive control. (D) Chromatin immunoprecipitation (ChIP) analysis of the binding to the p27 promoter by myc-tagged Cux proteins. Normal rabbit IgG was used as negative control of IP. Representative images are from PCR. Quantitative results are measured by real-time PCR. Data shown are mean ± SEM (n = 3 independent experiments). ** *P*<0.01, paired *t* test. (E–F) p21 decreased, while p27 and NCAM180 increased the dendritic arborization of cortical pyramidal neurons. Data shown are mean ± SEM (n≥30 for each condition). ** *P*<0.01, Student's *t* test. (G–H) Rescue of Cux1's effect on dendritic morphology of cortical pyramidal neurons by p27 and NCAM180. Data shown are mean ± SEM (n≥30 for each condition). ** *P*<0.01, ANOVA SNK test.

Cux1 functions as a transcriptional repressor for several genes in regulating cell proliferation and differentiation [1], [2]. Among them, the cyclin-dependent kinase inhibitors p21^Cip1^, p27^Kip1^, and the neural cell adhesion molecule (NCAM) were reported to regulate cytoskeleton dynamics in postmitotic neurons and were possibly required by Cux1 to regulate dendritic morphology of cortical pyramidal neurons [17], [18]. First, RT-PCR experiment found very low levels of p21 in our cultured cortical neurons at DIV3 and in the E16 (embryonic day 16) cortical tissue, unlike the high level of p21 in the P1 (postnatal day 1) thalamus (Figure 4C). Therefore, it's difficult to examine the effect of Cux1 overexpression on p21 in cortical neurons. Next, we analyzed the regulation of p27 and NCAM by Cux proteins in cortical neurons with RT-PCR. Both Cux1 and Cux2 could down-regulate the expression of NCAM (Figure 4A). In contrast, p27 was down-regulated by Cux1 only (Figure 4A). The down-regulation of p27 by Cux1, but not Cux2, was further confirmed by Western blotting (Figure 4B).

If one gene is repressed by Cux1 and required for Cux1 to regulate dendritic morphology, it should increase the dendritic complexity when overexpressed in cortical pyramidal neurons. We thus examined the effects of overexpressing p21, p27, and one of the three NCAM isoforms, NCAM180, which is predominantly expressed in neurons [19], on the dendritic morphology of cortical pyramidal neurons in culture. Comparing to the control, overexpression of p21 reduced the dendritic complexity, while overexpression of p27 and NCAM180 increased the dendritic complexity (Figure 4E–F). Thus, p27 and NCAM180 have stronger possibility than p21 to be the downstream mediator for Cux1 in controlling the dendritic morphology of cortical pyramidal neurons.

To investigate whether p27 and NCAM mediate the regulation of dendritic morphology by Cux1, we co-transfected p27 or NCAM180 with Cux1 in cultured cortical pyramidal neurons. When NCAM180 and Cux1 were co-expressed, the dendritic complexity was still decreased, and it did not have significant difference with Cux1 overexpression alone (Figure 4G–H). In contrast, co-expression of p27 with Cux1 increased the dendritic arborization level, and the dendritic complexity was significantly different from overexpression of Cux1 alone (Figure 4G–H). Thus, p27, but not NCAM, can compensate the reduction of dendritic arborization caused by Cux1 overexpression.

**Figure 5:**
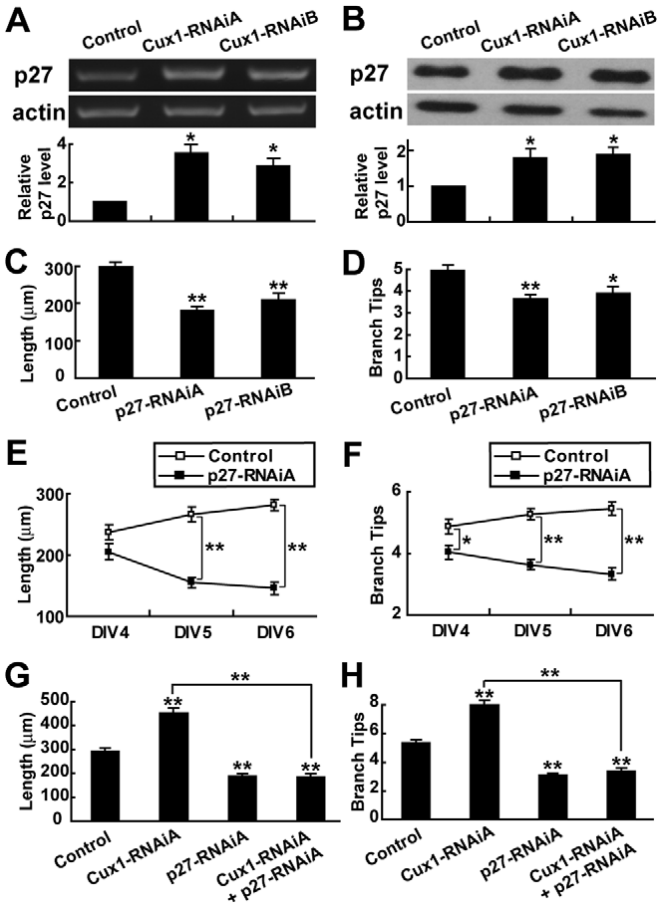
Modulation of p27 by RNAi-treatment of Cux1. (A–B) Increase of p27 expression level by transfection of Cux1-RNAi plasmids into cultured cortical neurons, as shown by RT-PCR (A) and Western blotting (B). Representative images in (A) are semi-quantitative PCR. Quantitative results in (A) are measured by real-time PCR. Data shown are mean ± SEM (n = 3 independent experiments for each condition). * *P*<0.05, paired *t* test. (C–D) Reducing the expression of p27 by RNAi simplified the dendritic complexity of cortical pyramidal neurons in culture. Data shown are mean ± SEM (n≥30 for each condition). * *P*<0.05, ** *P*<0.01, Student's *t* test. (E–F) Time-course of dendritic complexity change after p27-RNAiA treatment. Three time points were examined: DIV4, 5, and 6. Data shown are mean ± SEM (n≥30 for each condition). * *P*<0.05, ** *P*<0.01, Student's *t* test. (G–H) Rescue of Cux1-RNAiA's effect on dendritic complexity of pyramidal neurons by p27-RNAiA. Data shown are mean ± SEM (n≥30 for each condition). ** *P*<0.01, ANOVA SNK test.

To further examine whether p27 is required for Cux1 to regulate dendritic morphology, we constructed two RNAi constructs for p27, p27-RNAiA and p27-RNAiB, which were reported to be effective [20], [21], and transfected them into cultured cortical pyramidal neurons. Both of them decreased the dendritic complexity (Figure 5C–D). This effect followed similar time-course with Cux1-overexpression results (Figure 5E–F). RNAi treatments of Cux1 increased the p27 expression level (Figure 5A–B). Furthermore, p27-RNAiA could prevent the increase of dendritic complexity caused by Cux1-RNAiA (Figure 5G–H). Finally, consistent with a direct suppression of p27 expression by Cux1, chromatin immunoprecipitation (ChIP) analysis showed that Cux1 directly bond to the p27 promoter in cultured cortical neurons, while Cux2 had a much weaker affinity to the p27 promoter (Figure 4D). In summary, these results suggest that p27 is a direct target of Cux1 and is required by Cux1 to regulate dendritic morphology in cortical pyramidal neurons.

### RhoA mediates Cux1- and p27-induced dendritic morphology changes of cortical pyramidal neurons

**Figure 6:**
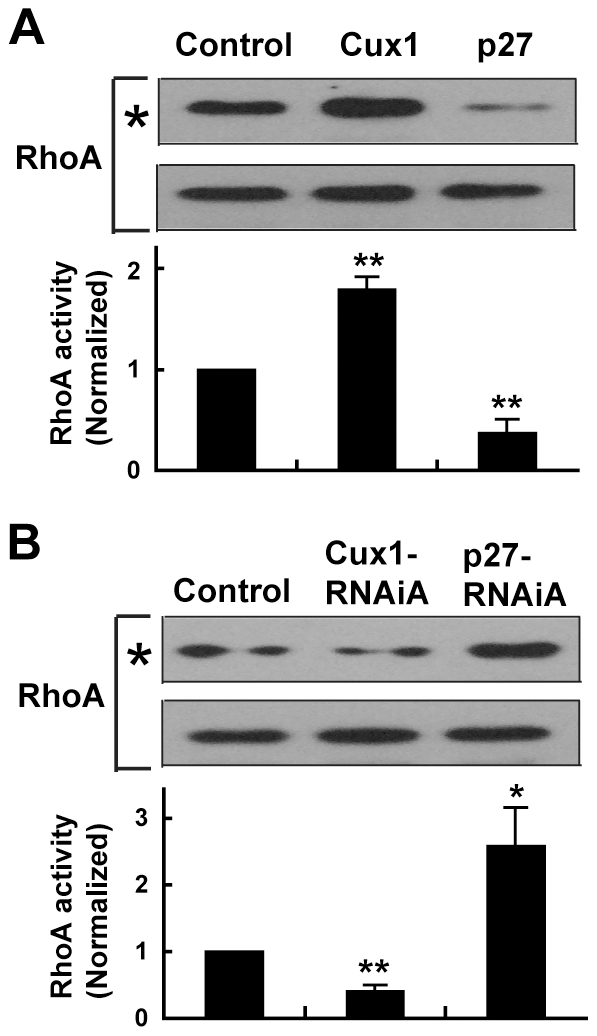
Regulation of RhoA activity by Cux1 and p27. Cux1 or p27-RNAiA increased RhoA activity, while p27 or Cux1-RNAiA decreased RhoA activity in cultured cortical neurons. Active RhoA was pulled down by GST-RBD and are indicated by asterisks. Protein samples of 1/10^th^ of total lysates were shown to indicate protein loading (bottom). Data shown are mean ± SEM (n = 4 independent experiments). * *P*<0.05, ** *P*<0.01, paired *t* test.

**Figure 7:**
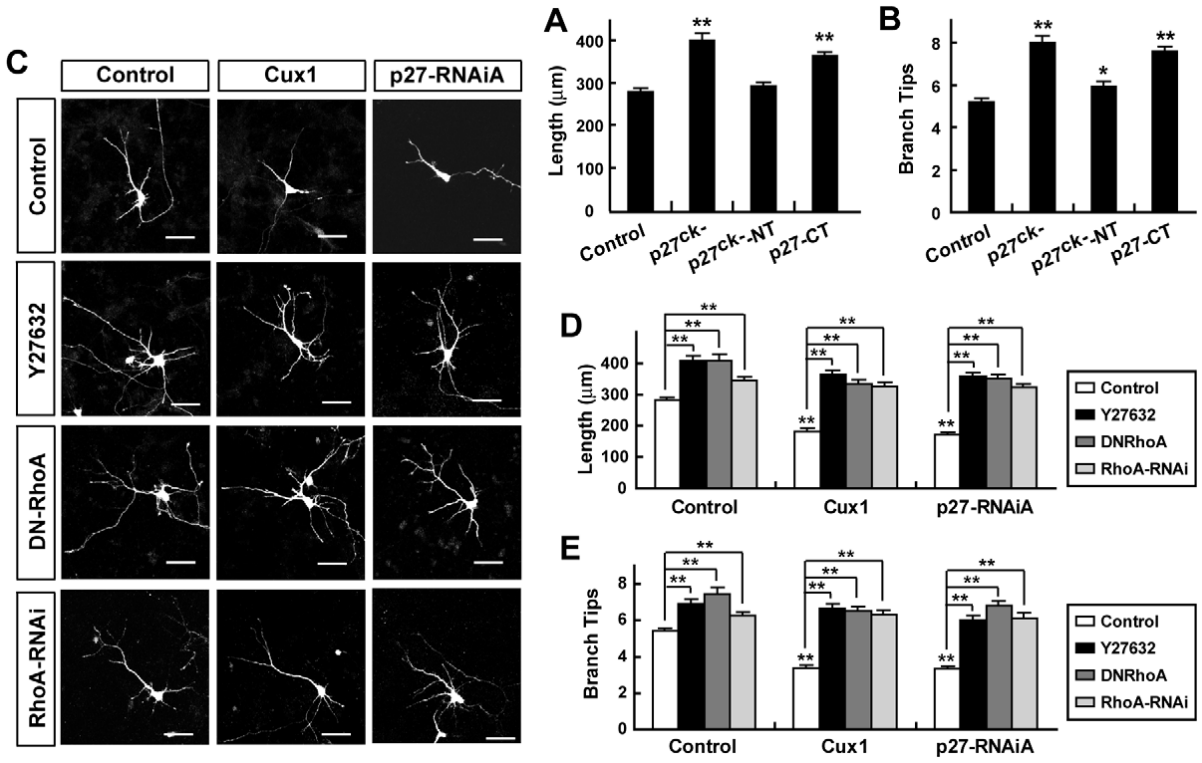
RhoA mediates Cux1- and p27-induced dendritic morphology changes of cortical pyramidal neurons. (A–B) Both p27^ck-^ (p27 deficient in the interaction with cyclins and Cdks) and p27-CT (C-terminal half of p27) could increase the dendritic arborization of cortical pyramidal neurons, while p27^ck-^-NT (N-terminal half of p27^ck-^) could only slightly affect the dendritic arborization. Data shown are mean ± SEM (n≥30 for each condition). * *P*<0.05, ** *P*<0.01, ANOVA SNK test. (C–E) Simplification of dendritic complexity of cultured cortical pyramidal neurons induced by either Cux1-overexpression or p27-RNAi could be prevented by treatment with 30 µM Y27632, co-expression of DN-RhoA, or co-expression of RhoA-RNAi. Data shown are mean ± SEM (n≥30 for each condition). ** *P*<0.01, ANOVA SNK test.

To further understand how Cux1 and p27 influence dendritic development, we examined whether the small GTPase RhoA, which acts downstream of p27 during neuronal migration and differentiation [22], could mediate the regulation of dendritic morphology by Cux1 and p27. First, we found that Cux1 and p27 could regulate RhoA activity in cultured cortical neurons. Overexpression of Cux1 or RNAi-treatment of p27 increased the level of active RhoA, while RNAi-treatment of Cux1 or overexpression of p27 decreased it (Figure 6). To determine if the region of p27 that is critical for RhoA regulation, rather than its Cdk inhibition function is actually relevant in control of dendritic outgrowth, we constructed the cyclin- and Cdk-specific interaction mutant of p27 (p27^ck-^), as previously described [22]. This mutation could still increase the dendritic complexity of cortical pyramidal neurons (Figure 7A–B). In addition, p27-CT (C-terminal half of p27), which mediates the interaction with RhoA [23], could also increase the dendritic arborization of cortical pyramidal neurons, contrary to the mild effect of p27^ck-^-NT (N-terminal half of p27^ck-^) (Figure 7A–B). Treatment of cultured cortical neurons with 30 µM Y27632, a specific inhibitor of Rho kinase, promoted dendritic arborization (Figure 7C–E), as previously reported [24]. When it was applied immediately after plasmid transfection, Y27632 could prevent the simplification of dendritic morphology induced by either overexpression of Cux1 or RNAi-treatment of p27 (Figure 7C–E). Furthermore, overexpression of dominant negative RhoA (DN-RhoA) or RNAi-treatment of RhoA could also rescue the phenotype of Cux1-overexpression and p27-RNAi (Figure 7C–E). These results suggest that RhoA, an important regulator of dendritic arbor growth [25], may mediate the regulation of dendritic complexity by Cux1 and p27.

## Discussion

As one of the most important features of neurons, the development of dendrites is regulated by both extrinsic and intrinsic factors [26]. Several transcription factors have recently been reported to control dendritic outgrowth of cortical pyramidal neurons. For example, the calcium-dependent nuclear transactivator CREST-deficient mice had severely compromised dendritic arborization of cortical layer V pyramidal neurons [27]. The transcription factor Zfp312 is selectively expressed by deep layer subcortical projection neurons, and knocking down its expression with RNAi altered their dendritic morphology [28]. The bHLH transcription factor Neurogenin-2 is both necessary and sufficient for specifying the unipolar dendritic morphology of cortical pyramidal neurons [29]. Here we identify the homeodomain transcription factor Cux1 as a negative regulator of the dendritic morphology of cortical pyramidal neurons. Interestingly, another homeodomain transcription factor, Phox2a, was shown to have regulatory cross-talk with Cux1 and activate the transcription of NCAM and p27 [4], [30]. This raises the possibility that Phox2a may also have the ability to control dendritic development.

Both Cux1 and Cux2 are homologues of the *Drosophila* Cut gene and have similar protein structure and the transcriptional suppressor activity. However, increasing evidences suggest that they are differentially regulated and have different functions. Cux1 is expressed in the brain and all major internal organs except liver, while Cux2 is expressed primarily in the nervous system [5], [6]. In the developing cerebral cortex, Cux1 does not have the same highly SVZ-restricted expression pattern as Cux2 [8]. In *Reeler* mutant mice, Cux1 distribution is correlated with the inverted cortex to the deep layers, whereas Cux2 is abnormally distributed throughout the cortical layers [10]. In contrast to Cux1, all combinations of Cux2 DNA-binding domains exhibit fast DNA binding kinetics [9], [31], [32]. Furthermore, this study shows that Cux1, not Cux2, is able to regulate the dendritic morphology of cortical pyramidal neurons. In addition, their unique homologue in *Drosophila*, Cut caused extensive dendrite branching when overexpressed in da sensory neurons [14], rather than the decreasing of dendritic arborization of cortical pyramidal neurons by Cux1 overexpression found in this study. Such divergence of Cux distribution and function may reflect the requirements of Cux genes in various signaling pathways by regulating the transcription of different genes and associating with different co-factors during transcription within varied cell types. Therefore, more detailed studies are required to better understand their functions in the future.

Although it has been suggested that the binding of Cux1 to the target promoters is diminished or even completely absent in some terminally differentiated cells [33], [34], the persistent expression of Cux1 in terminally differentiated cells of many tissues in the murine suggests a role of it in those tissues, as that of Cut in differentiated cells in *Drosophila* [8], [10], [14]. Our results support this idea by showing that Cux1 regulates the dendritic arborization of cultured cortical pyramidal neurons. As a factor restrictively expressed in upper cortical layers, Cux1 may thus contribute to the development of cortical circuitry by preventing the overgrowth of dendrites in upper layer pyramidal neurons. Numerous experiments suggest that neuronal activity plays a critical role in dendrite development and transcriptional regulation has been shown to mediate this process (reviewed in reference [35]). It would be of interest to examine whether Cux1 and its transcriptional activity can be regulated by neuronal activity and in hence contribute to the activity-dependent regulation of dendritic morphology during early postnatal development.

p27 is known to be the most important cyclin-dependent kinase inhibitor for cerebral cortex development [36]. Transgenic mice of full-length Cux1 and the short form p75-Cux1 were reported to have reduced expression of p27 in kidney, and Cux1 can directly interact with the p27 promoter and inhibit its activity [37], [38], [39]. Consistent with those reports, we found that Cux1 directly binds to the p27 promoter and reduces p27 level in cultured cortical neurons. We also found that p27 is the major downstream signaling molecule for Cux1 to control the dendritic complexity of cortical pyramidal neurons. Furthermore, p27 regulates the small GTPase RhoA [40]. p27 interacts with RhoA, and prevents RhoA activation by interfering its binding to GEFs [23]. p27 can also stabilize Neurogenin2, a transcriptional suppressor of RhoA [22], [29]. In general, RhoA activity is associated with the inhibition of dendritic arbor growth [25], [41]. Interestingly, we found that both Cux1 and p27 can regulate the activity of RhoA, and suppression of RhoA activity can prevent the simplification of dendritic morphology induced by either overexpression of Cux1 or RNAi-treatment of p27. Therefore, it is likely that during the development of cortical pyramidal neurons, RhoA may mediate the signaling from Cux1 to p27 to the regulation of cytoskeleton structure and dendrite development.

## Materials and Methods

### Animals

For all experiments we used Sprague-Dawley (SD) rats provided by SLAC Laboratory Animal Co., Ltd. Animal experiments were conducted under the guidelines of the Bioethics Committee of the Institute of Neuroscience at the Shanghai Institute for Biological Sciences and Chinese Academy of Sciences, with the Approval Number NA-060410.

### Gene cloning and plasmid construction

Plasmids were prepared with the Endo Free plasmid purification kit (Qiagen). The complementary DNA encoding rat Cux1 and Cux2 were obtained by PCR from P7 rat brain cDNA library. Cux1 ORF was divided into four fragments (1000∼1800 bp) for easier cloning and sequencing. The four fragments were cloned into pGEM-T Easy vector (Promega) and linked together by restriction enzyme cut and ligation to form a complete ORF of Cux1. Similarly, rat Cux2 ORF was divided into three fragments (1300∼2000 bp) and linked. Rat p21 was cloned from P7 thalamus cDNA library with the following primers: 5′-ATGTCCGATCCTGGTGATGTC-3′ and 5′-GGGCACTTCAGGGCTTTCTCT-3′. Rat p27 was cloned from P7 brain cDNA library with the following primers: 5′-TTACCATGTCAAACGTGAGAGTGT-3′ and 5′-CACTTACGTCTGGCGTCGAA-3′. Rat NCAM180 was obtained by PCR from the pRSV-NCAM180 construct provided by P. Maness with the 5′-ATGCTGCGAACTAAGGATCTCATC-3′ and 5′-TCATGCTTTGCTCTCATTCTCTTTC-3′ primers. DN-RhoA (N19) was obtained by PCR from construct from G. Bokoch with the 5′-ATGGCTGCCATCAGGAAGA-3′ and 5′-CTTCACAAGATGAGGCACCC-3′ primers. Deletions of Cux1 were cloned by PCR from pGEM-T Easy-Cux1. Mutation of p27 deficient in the interaction with cyclins and Cdks (p27^ck-^) was generated by PCR according to the literature [42]. p27^ck-^-NT (N-terminal half of p27^ck-^) and p27-CT (C-terminal half of p27) were constructed as described previously [22]. All of them were constructed into pCAGGS-IRES-EGFP vector to co-express them with EGFP under the control of the CAG promoter. To examine the expression and subcellular localization of Cux1 deletions, all of them were cloned into pCAG-EGFPN vector to fuse an EGFP tag at the C-terminal.

To construct the RNA-interference vectors, oligonucleotides targeting two distinct regions in the rat Cux1 coding sequence (Cux1-RNAiA: 5′-AGATGTCCACCACCTCAAA-3′; Cux1-RNAiB: 5′-AAGAAGAACACTCCAGAGGATTT-3′), the rat p27 coding sequence (p27-RNAiA: 5′-AAGCACTGCCGAGATATGGAA-3′; p27-RNAiB: 5′-GAAGCGACCTGCGGCAGAA-3′), the rat Cux2 coding sequence (Cux2-RNAiA: 5′-GCAGCATTCCTGAGTGTTT-3′; Cux2-RNAiB: 5′-TGGCCAACTTGAACAGTAT-3′), and the rat RhoA coding sequence (RhoA-RNAi: 5′-AAGGCAGAGATATGGCAAACA-3′) [43] were inserted into the pSuper.basic vector (Oligoengine) according to the manufacturer's instructions.

### Primary culture of cortical neurons

E16/17 cortical cells from SD rats were cultured as previously described [44]. The cortex was dissected in ice-cold HBSS (6.5 g/L glucose), digested with 0.25% Trypsin for 10 min at 37°C and dissociated into single cells by gentle trituration. Cells were plated on 35-mm culture dishes coated with 1 mg/ml poly-D-lysine at high density (6×10^6^ cells per dish) in DMEM (Invitrogen) with 10% fetal bovine serum for four hours, then changed to Neurobasal medium (Invitrogen) supplemented with B27 (Invitrogen), 0.5 mM L-glutamine, and penicillin/streptomycin.

### Transfections

Primary cultures of cortical neurons for morphometry analysis and immunofluorescence *in vitro* were transfected by a modified calcium phosphate transfection procedure [44]. For each culture dish, 3–5 µg of experimental plasmid was used to transfect cells at DIV3. The precipitate (60 µl) was added dropwise to the cells in 1 ml DMEM, and followed by a 20–30 min incubation. Unless indicated, cells were cultured to DIV6 before fixation.

Primary cultures of cortical neurons for RT-PCR, Western blotting, ChIP, and RhoA activity assay were transfected using Amaxa rat neuron nucleofector kit (Amaxa Biosystems) before being plated into 35-mm or 60-mm dishes, according to the manufacturer's instructions. Cells were cultured for two days before use.

### RT-PCR

The semi-quantitative RT-PCR to detect the RNAi efficiency and the relative mRNA levels of different genes was done as described [45]. Total RNA was extracted with Trizol reagent (Invitrogen). 5 µg of total RNA each was converted to cDNA with the RevertAid First Strand cDNA Synthesis kit (MBI Fermentas), and PCR was done with 1 µl cDNA each in 20-µl reactions (MJ Research). Primers and cycles used to amplify different genes were listed below. Primers for beta-actin were 5′-AACCGTGAAAAGATGACCCAGAT-3′ and 5′-TAATGTCACGCACGATTTCCCT-3′ for 25∼26 cycles. Primers for Cux1 were 5′-ATTGATGTTCCAGATCCCGTAC-3′ and 5′-CTCGTTCAAGGTCAGTCATAATCA-3′ for 31 cycles. Primers for Cux2 were 5′-GGCAGCGGTTGTTTGGTG-3′ and 5′- GCCCGTATCGGCGTTTCA -3′ for 31 cycles. Primers for NCAM were 5′-CTCCATCCACCTCAAGGTCTTCG-3′ and 5′-AGGGTCAGGGAGGACACACGAG-3′ for 30 cycles. Primers for p27 were 5′-GGTGCCTTCAATTGGGTCTCA-3′ and 5′-GGCTTCTTGGGCGTCTGCT-3′ for 30 cycles. Primers for p21 were 5′-TGACCTGGGAGGGGACAAGAG-3′ and 5′-GGGCACTTCAGGGCTTTCTCT-3′ for 35 cycles. Conditions for the PCR reactions were: first 94°C for 3 min; then the corresponding cycles of 94°C for 30 s, 58∼64°C for 30 s, and 72°C for 15∼25 s; with an 8 min 72°C final extension. Real-time PCR was conducted in the ABI PRISM 7000 Sequence Detection System (Applied Biosystems) with the SYBR Premix Ex Taq kit (TaKaRa) according to the manufacturers' instructions. The reactions were done in the presence of 5′-AAGACTAGCACCGTCATCAACTG-3′ and 5′-GCTTCCACGCCGTCACAAC-3′ primers for Cux1; 5′-GGCAGCGGTTGTTTGGTG-3′ and 5′-GCCCGTATCGGCGTTTCA-3′ primers for Cux2; 5′-GAGCAGTGTCCAGGGATGAG-3′ and 5′-CCACAGTGCCAGCATTCG-3′ primers for p27; 5′-CACCATCTACAACGCCAACA-3′ and 5′-ACATCACAGACAATCACAGCATC-3′ primers for NCAM; 5′-AGATTACTGCCCTGGCTCCTAG-3′ and 5′-CATCGTACTCCTGCTTGCTGAT-3′ for actin. Amplification cycle differences between experimental groups were calculated, corrected by actin as internal control, and converted to relative expression levels of corresponding genes. All the primers were designed to be located on different exons of the corresponding genes to eliminate the interference of residual chromosome DNA.

### Chromatin immunoprecipitation (ChIP)

ChIP assay was performed as previous described [46] with some modifications. Briefly, cultured cortical neurons were transfected with plasmids encoding myc-tagged Cux1 or Cux2 protein, or the empty vector as control. Cells were cross-linked with fresh 1% formaldehyde for 10 min at room temperature, and the reaction was terminated with an excess of glycine. Chromatin was sonicated into an average size of 250–350 bp and was immunoprecipitated with anti-myc polyclonal antibody (Cell Signaling) or a control normal rabbit IgG (Santa Cruz). Co-precipitated chromatin was analyzed by PCR for the presence of the p27 promoter region using the 5′-GAGTCGTCAGTCCTGGTTCCT-3′ and 5′-GGGAGGGTAGGCGAAAGAT-3′ primers, generating a 182-bp product. The PCR conditions consisted of 94°C for 3 min, followed by 35 cycles of 94°C for 20 sec, 61°C for 15 sec and 72°C for 15 sec, with a final extension of 72°C for 5 min. The PCR results were cloned and sequenced to verify the specificity of PCR. Quantification of ChIP results was done by real-time PCR with the same primers.

### RhoA activity assay

RhoA activity was measured as described previously [47]. Briefly, lysates of cortical neurons transfected with various plasmids were preincubated with glutathione-Sepharose 4B beads (Amersham Biosciences) at 4°C for 1 h with constant rocking to remove the nonspecific binding. The supernatants were then incubated with the bacterially produced glutathione S-transferase (GST)-fused Rho-binding domain of Rhotekin (GST-RBD; 20 µg) bound to glutathione-coupled Sepharose beads at 4°C for 1 h with constant rocking. The beads were washed five times with cell lysis buffer at 4°C, then the proteins were eluted in SDS sample buffer and analyzed for bound RhoA by Western blotting using the rabbit antibody against RhoA (Cell Signaling). Protein samples of one-tenth of total lysates were shown to indicate similar loading.

### Immunofluorescence

Cell cultures were fixed with 3% paraformaldehyde and 2% glutaraldehyde in phosphate-buffered saline (PBS) for 10 min. For Propidium iodide (PI) staining, fixed cells were stained with 2 µg/ml PI for 3 min at room temperature. Images were captured with the FLUOVIEW FV1000 confocal system (Olympus).

### Western blotting

For Western blotting, 1/10th of the samples were loaded each lane in the Mini-PROTEAN 3 System (Bio-Rad). Immunoblotting was performed as described [47]. Primary antibodies used were mouse anti-actin (Chemicon, 1∶10 000), and mouse anti-p27 (BD Biosciences, 1∶1 000). Secondary antibodies of HRP-conjugated goat-anti-mouse (Bio-Rad, 1∶10 000) were used and visualized with a chemiluminescent substrate (SuperSignal, Pierce). The relative signal intensities of at least three independent results for each Western blotting experiment were measured with the software ImageQuant Ver.5.0 (Molecular Dynamics).

### Morphometry analysis and statistics

Pyramidal neurons in culture were defined as cells with a pyramidal cell body and one prominent apical dendrite at its apex, with an overall morphology typical for cortical pyramidal neurons, as described [44], [48]. Neurons were reconstructed and scored for the total dendritic branch length (Length) and the total dendritic branch tip number (Branch Tips) with the software FV10-ASW Ver.1.4a (Olympus). Axons were identified on the basis of the characteristic morphology and relatively longer length. Statistical significances were determined using the two-tailed Student *t* test, paired *t* test, or the ANOVA Student-Newman-Keuls (SNK) test, as appropriate.
